# Improved 2D Ground Target Tracking in GPS-Based Passive Radar Scenarios

**DOI:** 10.3390/s22051724

**Published:** 2022-02-23

**Authors:** Pedro Gomez-del-Hoyo, Nerea del-Rey-Maestre, María-Pilar Jarabo-Amores, David Mata-Moya, María-de-Cortés Benito-Ortiz

**Affiliations:** Signal Theory and Communications Department, Polytechnic School, University Campus, University of Alcalá, 28805 Madrid, Spain; pedrojose.gomez@uah.es (P.G.-d.-H.); nerea.rey@uah.es (N.d.-R.-M.); david.mata@uah.es (D.M.-M.); cortes.benito@uah.es (M.-d.-C.B.-O.)

**Keywords:** passive radar, GPS, array, ground target, GNSS

## Abstract

Global Positioning System (GPS) satellites offer promising opportunity for Passive Radar systems due to their global coverage and the availability of multiple satellites throughout the world. However, their low power at ground level limits system coverage. In this paper, a GPS based Passive Radar which exploits a single illumination source, and uses digital array processing for ground targets localization is presented. To face signal power problems, a processing scheme combining reconstructed reference signals, adaptive filtering techniques and spatial filtering is implemented. Conventional beamforming techniques are used to increase the level of the target echo before the detection stage, and high resolution DoA estimation techniques are applied to estimate targets azimuth. Ground target localization in local Cartesian space is performed taking into account the system geometry, range and azimuth information. Both synthetic and real radar data are used to analyse system operation. During the measurement campaign, a cooperative vehicle was used for validation purposes. Results confirm that ground targets detection and localization are feasible using a single GPS transmitter.

## 1. Introduction

A Passive Radar (PR) is defined as a set of techniques that use non-cooperative signal sources, denominated Illuminators of Opportunity (IoO), to detect and extract characteristics (velocity, position) from the targets inside an Area of Interest (AoI) [1]. Due to the absence of a dedicated transmitter, PRs present several advantages as reduced design and deployment costs, the possibility of using commercial of the shell components, and not requiring frequency allocation. In the radar literature, multiple PR demonstrators have been developed using different terrestrial communication systems, such as analog and digital broadcasting [2,3], digital television broadcasting [4], and mobile communication systems [5].

There is a growing interest in exploiting satellite platforms as IoOs due to the great diversity of constellations, signal waveforms and frequencies, which provide high availability, global coverage, and almost total invulnerability to natural disasters. Nevertheless, the large distance from the transmitter reduces signal power at ground level and compromises the PR performance. An important reduction of the ground coverage, although IoO availability is guaranteed along all the Earth surface, is expected in comparison with PRs which use ground-based illumination sources [6].

The satellite’s orbit determines the influence of the platform dynamic on the PR scenario. Geostationary orbits produce a negligible relative movement whereas medium and low Earth orbits produce variations in PR geometry which affect the acquired signal characteristics. Coherent processing of the reference and surveillance signals can be applied when the platform movement is negligible and the transmission and reception characteristics allow the direct isolation of the desired illumination signal such as in DVB-S based PRs [7,8]. More complex processing schemes should be applied in other cases, such as Global Navigation Satellite System (GNSS) based PRs, where the platform movement and the medium access technique require the isolation and tracking of the reference signal.

GNSS satellites are promising illumination sources for PRs because of the global coverage they provide, and the simultaneous availability of multiple transmitters anywhere in the world. GNSS constellations have been exploited for maritime target detection and imaging [9,10,11,12] and for aerial targets detection [13,14]. A GPS based PR for ground target detection was presented in [15]. In all cases, long integration times are used to address the low GPS power at ground level, and multistatic configurations are considered for target localization by means of multilateration. These localization techniques require the detection of a target from at least three transmitter-receiver pairs. Nevertheless, in the case of weak echo targets as the ground vehicles, the detection from multiple transmitter-receiver pairs can fail.

In this paper, a digital processing scheme is proposed for ground target passive localization by means of a single GPS transmitter. This work is a continuation of [16], where a study of the potentials of array surveillance antennas for ground target localization exploiting a single GPS satellite was presented. The civilian navigation signal at L1 band is selected as the illumination signal. A processing scheme based on the reconstruction of the reference signal is designed to tackle the limitations related to the low power of the received GPS signal. Adaptive filtering techniques based on the Extensive Cancellation Algorithm (ECA) are considered to reject the Direct Path Interference (DPI) and digital array processing schemes have been implemented to include the spatial integration gain in the detection stage. The ground target location is estimated applying high resolution Direction of Arrival (DoA) techniques and considering the bistatic reception configuration. The proposed localization scheme is validated with synthetic and real radar data acquired by PR demonstrator IDEPAR developed in the University of Alcalá [17].

The rest of the paper is organised as follows: in Section 2, the operation principle of a GPS based PR is detailed. The proposed digital processing architecture for ground target detection and localization is presented in Section 3, which is the Cartesian tracker stage detailed in Section 4. System localization performances are analysed in Section 5 with synthetic data. Section 6 presents the system validation with real GPS data acquisition, including a description of the selected radar scenario and the experimental results for cooperative and non-cooperative ground targets. Finally, the conclusions are remarked in Section 7.

## 2. System Operation Principle

**Figure 1:**
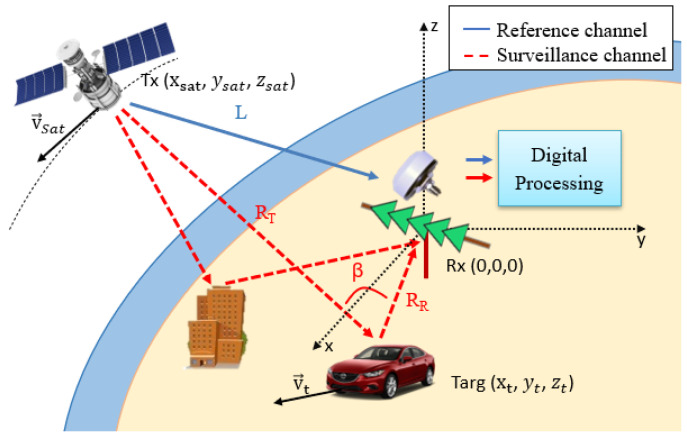
PR scenario based on satellite IoO, consisting of a hemispherical reference antenna to allow acquisition of all GPS signals in line of sight, and a surveillance array to acquire targets echoes.

The basic geometry of a PR based on a space-borne IoO is depicted in Figure 1. The acquisition system is usually composed of two independent channels: one is the reference channel, for acquiring the direct signal from the satellite, $s_{ref}\left(t\right)$; and the other is the surveillance channel, in charge of recording target echoes from the AoI, $s_{surv}\left(t\right)$.

A traditional PR bases its operation in the coherent processing of both acquired channels. After a preprocessing stage, that reduces DPI and Clutter interferences, a Cross Ambiguity Function (CAF) between the reference and surveillance signals is computed according to Expression (1).

(1) $$S_{CAF}\left[m,p\right]=\sum_{n=0}^{N-1}s_{ref}^{*}\left[n-m\right]\cdot s_{surv}\left[n\right]\cdot \exp^{-j2\pi \frac{p}{N}n}$$

The presence of a target echo in the surveillance signal gives rise to a local maximum at a bistatic range-Doppler shift pair (*m*,*p*) related to its location and movement with respect to the PR geometry.

In the specific case of GPS satellite based PRs, a deeper analysis of the transmitted signals and PR geometry should be carried out to allow the correct definition of the PR processing scheme.

GPS operation is based on the continuous and simultaneous transmission of GPS signals from 24 satellites in a medium earth orbit (MEO), in three different frequency bands (L1, L2, L5). In this work, civil signals in the L1 band are considered as illumination sources. All GPS L1 signals share the same carrier frequency in a Code Division Multiple Access (CDMA) method requiring a signal reconstruction stage to isolate the selected reference signal. In addition, the platform location in an MEO generates a time variant geometry that should be considered in reference signal reconstruction and PR measurement interpretation. Both issues are considered in the following subsections.

### 2.1. GPS Passive Radar Geometry

The PR configuration considered in this work is depicted in Figure 1. A local Cartesian coordinate system centred in the PR location is selected to define the system geometry and to generate the position of the targets. A single hemispherical antenna with right hand circular polarization was considered to acquire the direct signal from the line of sight GPS satellites and a linear array composed of 5 single vertical polarized elements was selected for the surveillance channel. This configuration provides the following target information in a 3D vector: bistatic range and Doppler shift from coherent signal processing, and azimuthal DoA after applying digital beamforming techniques.

The bistatic range *m* is determined by the difference of the path lengths travelled by the wave received directly from the illuminator and the wave scattered by the target, as expressed in (2).

(2) $$m=R_{T}+R_{R}-L$$

where $R_{T}$, $R_{R}$, and *L* are the distances from the satellite to the target, from the PR to the target, and from the satellite to the radar, respectively. Due to satellite movement, the whole parameter set has a time dependence and could be different in each processing interval.

The second PR measurement is the Doppler shift, *p*, which is the frequency difference between the reference signal and target echo. In a moving transmitter environment, a Doppler frequency appears in both acquired signals: in the reference one due to satellite movement; and in the radar echo due to both transmitter and target movements. To calculate the Doppler shift, the projections of the current velocity vector on all signal paths are considered:

(3) $$p=-\frac{\left({\vec{v}}_{t}-{\vec{v}}_{sat}\right)\cdot {\vec{R}}_{T}+{\vec{v}}_{t}\cdot {\vec{R}}_{R}+{\vec{v}}_{sat}\cdot \vec{L}}{\lambda }$$

where λ is the carrier wavelength, $\vec{x}\cdot \vec{y}$ represents the scalar product between vectors $\vec{x}$ and $\vec{y}$, and ${\vec{R}}_{R}$, $\vec{L}$ and ${\hat{R}}_{T}$ are the director vectors from receiver to target, from transmitter to receiver, and from transmitter to target, respectively.

Finally, the presence of a surveillance array allows us to estimate the target’s azimuth ϕ, which is related to the target location as $tan\left(\phi \right)=y_{t}/x_{t}$.

### 2.2. GPS Passive Radar Signal Processing

In each GPS transmitter, the navigation message of the signal civil part, $N_{M}\left(t\right)$, is encoded by a unique Pseudo-Random Noise (PRN) code with a repetition cycle of 1 ms, CA(t), to generate a spread spectrum signal, and placed in a common carrier frequency, $f_{c}$, which depends on the GPS band.

**Figure 2:**
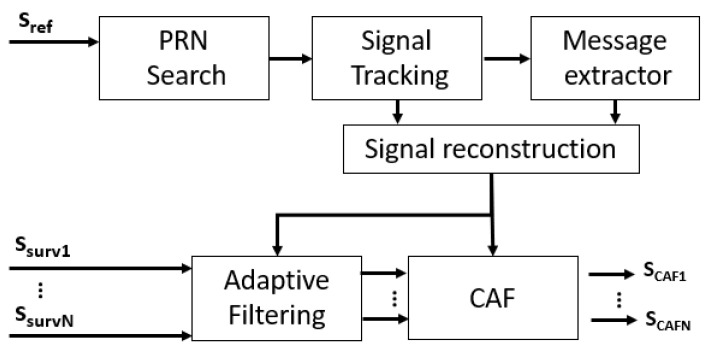
Signal processing schema based on GPS signal regeneration, adaptive filtering and CAF.

The civilian signal transmitted in the L1 band is described by (4), where i is the satellite PRN index, and Ap is the amplitude factor. Since the carrier frequency is shared by all GPS satellites, the PR acquired signal is composed of signals from multiple illumination sources. This problem and the low received power in reference and surveillance channels, make the use of a traditional PR processing schema in which the acquired reference signal is directly employed in the coherent processing stage impossible. To solve both problems, a PR processing scheme based on the use of reconstructed reference GPS signals was selected. The GPS based processing chain employed in this paper is depicted in Figure 2. Firstly, the reference channel is used to find, track and regenerate the available GPS signals, providing signal source differentiation.

(4) $$SAT_{i}\left(t\right)=Ap_{i}\cdot N_{Mi}\left(t\right)\cdot CA_{i}\left(t\right)\cdot cos\left(2\pi f_{c}t\right)$$

The acquired direct path signal, $s_{i}$, after quadrature demodulation, excluding constant amplitude and phase terms, can be modelled as (5), where the sub-index *i* represents the PRN associated with the GPS satellites in line of sight from PR location. Each individual GPS signal will be delayed in time, $\tau_{i}\left(t\right)$, and shifted in frequency, $f_{D,i}\left(t\right)$, due to the space-platform distance and movement, respectively. Due to the orbital movement, the delay and Doppler shift are time dependent, so a signal tracking process is required.

(5) $$s_{i}\left(t\right)=N_{Mi}\left(t-\tau_{i}\left(t\right)\right)\cdot CA_{i}\left(t-\tau_{i}\left(t\right)\right)\cdot exp\left(j2\pi f_{D,i}\left(t\right)t\right)$$

The GPS receiver software available in the literature allows the GPS signal search and tracking process necessary for signal reconstruction [18]. In this work, the satellite presence and the first time delay and Doppler frequency estimations were carried out using a circular correlation method. In the signal tracking stage, a combination of a digital second order Phase Locked Loop (PLL), and a Delay Locked Loop (DLL), is implemented in intervals of 1ms to provide the Doppler frequency and time delay tracking of the GPS signals. Once the Doppler frequency and time delay are estimated, the navigation message is extracted and the whole reference signal can be reconstructed.

Before CAF generation, direct path interference components from all available satellites are filtered in the surveillance signals acquired by the different single radiating elements of the surveillance antenna array, using ECA filters [19].

The search stage ideally finds all GPS signals that are acquired on the reference channel. However, very low power echoes are expected from targets, and signals with low correlation levels compromise the performance of the PR. In this work, the target location will be obtained using a single satellite signal. Therefore, the coherent radar processing stage to generate the Range Doppler Maps (RDMs) is performed with the CAF taking as input the reconstructed reference signal associated with the most powerful received satellite, and the filtered surveillance signal.

## 3. Ground Target 2D Localization Scheme

In PRs, echoes received from targets are characterized by low power. For GPS-based PRs, the power budget is even more restrictive, because even the direct reference signal is at or below the noise level, which jeopardizes the detection and localization of small targets, such as terrestrial ones. To increase the power of target echoes and enable target localization, a two-stage spatial filtering approach is designed by taking advantage of a surveillance array system.

The proposed approach is based on [20] in which multiple beams were simultaneously generated in the frequency domain to perform target detection and bistatic tracking considering the spatial integration gain of conventional beamforming techniques. After the detection stage, a second spatial processing step, based on direction-of-arrival techniques, was applied to produce estimates of higher angular accuracy that can be used to obtain the location of targets in a local Cartesian space. In this work, the spatial processing scheme is slightly modified to avoid target tracking in the bistatic domain, and perform target localization directly by Cartesian tracking after target angular information extraction. In addition, the first orthogonal beamforming stage is complemented with overlapping beams to improve the SNR of the target echo over the entire coverage area.

**Figure 3:**
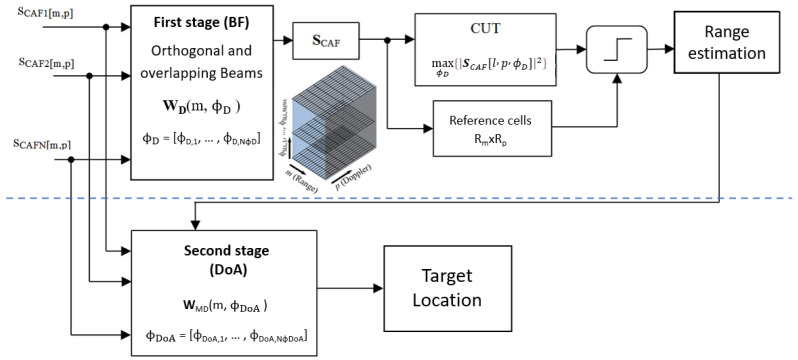
Proposed two-stage frequency-domain spatial filtering scheme.

The functional block of the proposed processing scheme is depicted in Figure 3 where two block groups can be differentiated: the first part of the scheme carries out target detection and parameters estimation in the bistatic domain taking into consideration the beamforming gain of conventional beamforming techniques applied to the RDMs of the individual antennas; on the second part high accuracy DoA techniques are applied to provide targets azimuth estimations and allow accurate target location in Cartesian space. The computation of individual CAFs, which are required as input for this processing scheme, has already been considered in Section 2.2, while the two-stage digital array processing and the target detection and localization algorithms are presented in the following subsections.

### 3.1. First Stage Spatial Filtering

This stage focuses on increasing the target echo power to enable detection of small targets in the range-Doppler domain, taking advantage of the beamforming gain in a ubiquitous radar configuration with a set of two simultaneous beams. The selected digital beamforming algorithm operates in the frequency domain after applying the CAF to the individual surveillance channels. This approach takes advantage of the inherent clutter and target mapping in the range-Doppler domain of the coherent processing stage to isolate the energy of each contributor [20].

**Figure 4:**
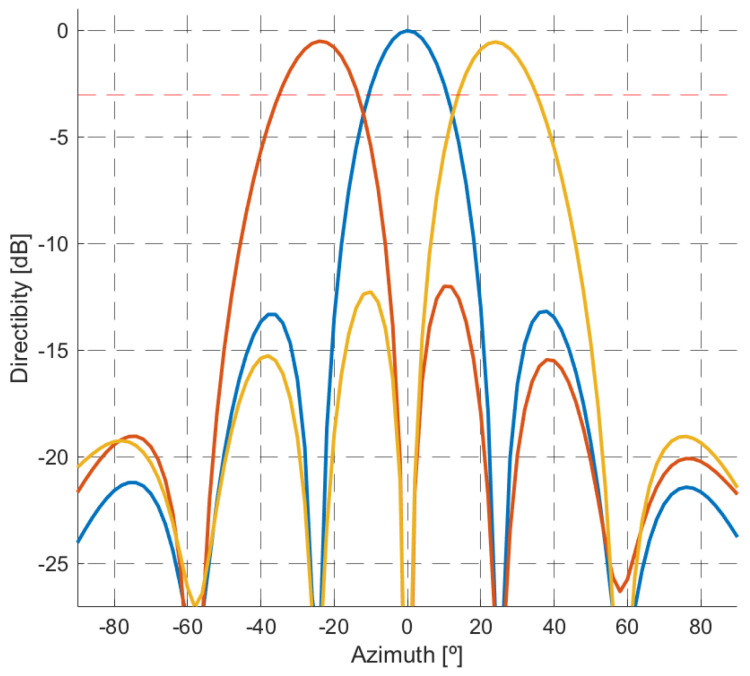
Set of three orthogonal beams for a five elements array.

- To form the first set of simultaneous beams, digital beamforming techniques were selected under the requirement of sidelobe level control to generate orthogonal beams along with the azimuth sector of the single radiating element. An iterative process was followed to define the *N* orthogonal beam steering angles, $\Phi_{SLL}=\left\{\phi_{SLL,1},\phi_{SLL,2},\ldots ,\phi_{SLL,N}\right\}$. The process starts with the first lobe steered to the broadside, then the steering direction of the adjacent lobes is adjusted to the first null of the initial beam. The process continues with the following adjacent lobes until the entire azimuthal sector of a single radiating element is covered.
- The orthogonal beams design procedure reduces the contribution to signal power in the current beam of targets whose DoAs are the steering directions of adjacent beams. However, the decrease in gain with respect to the maximum at the intersection points of the beams can be greater than 3 dB, negatively affecting the echoes of the targets in those directions (Figure 4). As the main objective of this first stage of spatial filtering is to improve target echoes SNR to allow their detection, beamforming gain losses should be minimized along the whole coverage area. Therefore, a second set of *N* − 1 steering angles, $\Phi_{LI}=\left\{\phi_{LI,1},\phi_{LI,2},\ldots ,\phi_{LI,N-1}\right\}$, was defined according to the crossing points of the previous orthogonal beams. Both steering angle sets are merged together in a steering vector to continue the design process, $\Phi_{D}=\left[\Phi_{SLL},\Phi_{LI}\right]$.
- The optimization problem was solved for each steering direction and Doppler shift pair ($\phi_{D,i}$,*p*) to compute the weight vector $w_{D}\left(p,\phi_{D,i}\right)$. Applying the weight vectors to the corresponding snapshots in the transformed domain (6), a three dimensional matrix, $S_{\mathrm{CAF}}\left[m,p,\phi_{D,i}\right]$ is obtained.
  (6) $$\begin{array}{c} S_{\mathrm{CAF}}\left[m,p,\phi_{D,i}\right]=w_{D}{\left(p,\phi_{D,i}\right)}^{H}\cdot s_{s}\left[m,p\right]\\ \;i=1,\cdots ,2N-1 \end{array}$$

### 3.2. Detection Stage

To carry out targets’ detection, a Cell Average-Constant False Alarm Rate (CA-CFAR) based detector is selected. To manage the 3-dimensional matrix, $S_{\mathrm{CAF}}\left[m,p,\phi_{D,i}\right]$, a windowing technique that defines one single Cell Under Test (CUT) for each [m,p] pair is designed. The CUT is selected as the one with the maximum power value from $s_{CAF}\left[m,p,\phi_{SLL,i}\right]$ in the $\phi_{SLL,i}$ dimension. The reference window spreads along the range-Doppler plane around the selected CUT. Guard cells are defined to reduce the impact of oversampling and extended targets in the threshold estimation.

The estimated location of targets in detection space is the centroid of positively detected points or the position of the maximum level. However, a more accurate estimation of the targets range can be obtained when the reference and surveillance signals are oversampled by taking advantage of the signal correlation characteristics [21]. An improved estimation of target range is obtained by approximating the GPS signal correlation with a parabola. In this paper, the bin with the maximum level and its four adjacent bins are used to calculate a second-order function that approximates the CAF in the range dimension. The target range is estimated as the range where the parabola has the maximum value.

**Figure 5:**
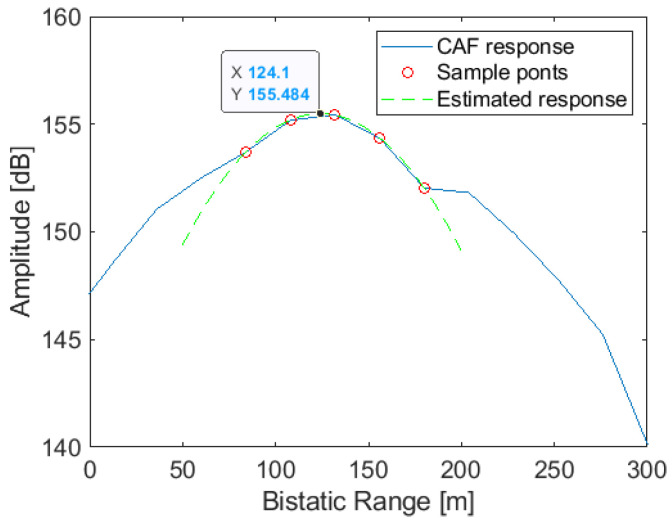
Target’s range estimation by means of 5 points around the maximum. The real target range is 125 m, while the maximum of the CAF is obtained at 132 m. The estimated range with this approach is 124.1 m.

Figure 5 shows the range profile of a punctual target at 125 m considering an L1 GPS signal with a sampling frequency of 12.5 MHz. The approximation with a parabola using five points is also depicted, showing an estimated range value (124.1 m), which is close to the maximum in the CAF. The estimation accuracy depends on the echo quality as a function of its SNR, as shown in Expression (7).

(7) $$\sigma_{m}=\frac{A}{\sqrt{\mathrm{SNR}}}\Delta m$$

where Δm is the range resolution and *A* is a scalar coefficient, typically in the interval (0.5,0.9).

### 3.3. Second Stage Spatial Filtering

At this stage, the azimuth of the detected targets is estimated using a high-accuracy DoA estimation technique. The accuracy of azimuth estimation was increased by generating a new set of steering angles $\Phi_{DoA}=\left\{\phi_{DoA,1},\phi_{DoA,2},\ldots ,\phi_{DoA,N_{\phi_{DoA}}}\right\}$ composed of a high number of steering directions with low azimuth increments between them. The new beamforming weights vector is calculated according to a maximum array directivity criteria, $w(p,\phi_{DoA})$. To produce the DoA estimation the Minimum Variance Distortionless Response (MVDR) algorithm is considered [20]. The estimated DoA is the angle ϕ where a maximum of the beamformer output spectrum is obtained. The beamformer spectrum is calculated with Expression (8).

(8) $$\begin{array}{c} S_{m,p}\left(\phi \right)=w{\left(p,\phi_{DoA}\right)}^{T}\cdot {\hat{R}}_{s_{s},s_{s}}\left(m,p\right)\cdot w\left(p,\phi_{DoA}\right)\\ {\hat{R}}_{s_{s},s_{s}}\left(m,p\right)=s_{s}\cdot s_{s}^{H} \end{array}$$

where ${\hat{R}}_{s_{s}s_{s}}\left(m,p\right)$ is the estimation of the instantaneous spatial covariance matrix for the snapshot $s_{s}\left(m,p\right)$ at target location (m,p).

### 3.4. Target Localization

The measurement vector provides information of the target’s bistatic range (*m*), and DoA (ϕ), as input parameters to estimate the target’s position in local Cartesian space. In addition, the target motion is expected to be parallel to the local Cartesian plane at a known altitude from the PR location, alt.

**Figure 6:**
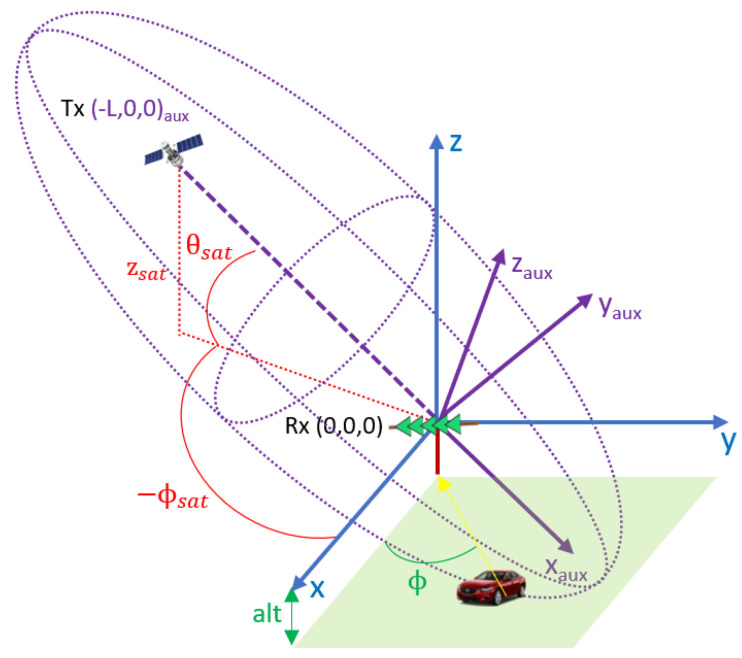
PR geometry: local Cartesian space (blue), auxiliary coordinate system (purple).

The measured bistatic range (*m*), defines a three-dimensional ellipsoid of revolution with the GPS satellite and the PR located at the foci. The equation of this ellipsoid is (13) in an auxiliary coordinate system, $\left\{x_{aux},y_{aux},z_{aux}\right\}$, which is obtained from the local Cartesian space by a first rotation on the *z*-axis with an angle $\phi_{rot}=\phi_{sat}+\pi$, followed by a second rotation on the *y*-axis according to the satellite’s inclination, $\theta_{sat}$. These two rotations place the position of the transmitter at (−L,0,0) in the auxiliary coordinate system as shown in Figure 6.

(9) $$\frac{{\left(x_{aux}+L/2\right)}^{2}}{a^{2}}+\frac{y_{aux}^{2}}{b^{2}}+\frac{z_{aux}^{2}}{c^{2}}=1$$

where a=(m+L)/2 is the length of the major semi-axis and $b=c=\sqrt{a^{2}-{\left(L/2\right)}^{2}}$ is the length of the minor semi-axis. The DoA of the target in the new coordinate system obtained after applying the first rotation is $\phi^{\prime }=\phi -\phi_{rot}$, whereas the target altitude remains invariant. The target positions fulfil the following equations:

(10) $$\begin{array}{cc} x^{\prime } & =y^{\prime }/tan\left(\phi^{\prime }\right)\\ z^{\prime } & =alt \end{array}$$

After applying the second rotation in the *y*-axis according to the inclination of the satellite, $\theta_{sat}$, the target positions in the auxiliary coordinate system have to satisfy the following equations:

(11) $$\begin{array}{cc} x_{aux} & =x^{\prime }cos\left(\theta_{sat}\right)+z^{\prime }sin\left(\theta_{sat}\right)=ey_{aux}+d\\ y_{aux} & =y^{\prime }\\ z_{aux} & =-x^{\prime }sin\left(\theta_{sat}\right)+z^{\prime }cos\left(\theta_{sat}\right)=fy_{aux}+g \end{array}$$

with $e=cos\left(\theta_{sat}\right)/tan\left(\phi^{\prime }\right)$, $d=altsin(\theta_{sat})$, $f=-sin\left(\theta_{sat}\right)/tan\left(\phi^{\prime }\right)$, and $g=altcos(\theta_{sat})$. By substituting Equations (11) in (9), and after several organisation operations, the second order equation shown in (12) is obtained.

(12) $$a^{\prime }y_{aux}^{2}+b^{\prime }y_{aux}+c^{\prime }=0$$

where:

(13) $$\begin{array}{cc} a^{\prime } & =\frac{e^{2}}{a^{2}}+\frac{1}{b^{2}}+\frac{f^{2}}{c^{2}}\\ b^{\prime } & =2\left(\frac{(d+L/2)e}{a^{2}}+\frac{fg}{c^{2}}\right)\\ c^{\prime } & =\frac{{\left(d+L/2\right)}^{2}}{a^{2}}+\frac{g^{2}}{c^{2}}-1 \end{array}$$

Solving (12) and substituting in (10), two possible target locations are obtained, after the first *z*-axis rotation. The ambiguous solution can be solved considering the target arrival angle $\phi^{\prime }$. Finally, the target location in the local Cartesian space is obtained by inverting the first rotation of the *z*-axis with expressions in (14).

(14) $$\begin{array}{cc} x & =x^{\prime }cos\left(-\phi_{rot}\right)-y^{\prime }sin\left(-\phi_{rot}\right)\\ y & =x^{\prime }sin\left(-\phi_{rot}\right)+y^{\prime }cos\left(-\phi_{rot}\right)\\ z & =z^{\prime }=alt \end{array}$$

### 3.5. Estimation of the Initial Target Velocity

Since targets of interest are ground vehicles and their movements are assumed to be confined to roads in the area of interest, an initial velocity estimate can be made by including external geographic information system data. The velocity vector can be estimated from the bistatic velocity of the targets ($v_{b}$), and the road orientation, with the resulting direction obtained by considering the previous location estimate. The target’s bistatic velocity is obtained from the measured Doppler shift, extracting the Doppler components from satellite movement, as expressed in (15).

(15) $$v_{b}=-\frac{p}{\lambda }+{\vec{v}}_{sat}\cdot {\vec{R}}_{T}-{\vec{v}}_{sat}\cdot \vec{L}$$

where ${\hat{R}}_{T}$ and $\hat{L}$ are the direction vectors from transmitter to target and receiver respectively, and ${\vec{v}}_{sat}$ the satellite velocity vector.

Therefore, the components of the target velocity vector $\left[v_{x},v_{y}\right]$ are computed according to the local orientation angle of the nearest road, γ. If tan(γ)=∞, the velocity vector is in the *y*-axis direction ( $\left[v_{x},v_{y}\right]=\left[0,v_{b}\right]$). Otherwise, the system of equations in (16) must be solved.

(16) $$\begin{array}{cc} \; & v_{b}=v_{x}\left(x_{Tx}+x_{Rx}\right)+v_{y}\left(y_{Tx}+y_{Rx}\right)\\ \; & tan\left(\gamma \right)=v_{y}/v_{x} \end{array}$$

where ($x_{Tx}$, $y_{Tx}$), and ($x_{Rx},y_{Rx})$ are the components of the direction vectors from the transmitter to the target location (${\vec{R}}_{T}$), and from the receiver to the target location (${\vec{R}}_{R}$).

After substitution and simplification, the components of the velocity vector can be calculated with (17).

(17) $$\begin{array}{cc} v_{x} & =\frac{v_{b}}{x_{Tx}+X_{Rx}+tan\left(\gamma \right)\left(y_{Tx}+y_{Rx}\right)}\\ v_{y} & =v_{x}tan\left(\gamma \right) \end{array}$$

## 4. Target Tracking

PR measurements are applied directly to a Cartesian tracking stage based on the Kalman filter [22], to obtain the target location. The application of the Kalman filter to target tracking is common in PR [23,24,25]. The core of the tracker is a recurrent prediction-correction process that is usually performed by stochastic filters. These filters use a recurrent prediction/correction process to estimate the state of the target taking into consideration a set of noisy measurements and a dynamic model that represents the target movement.

The performance of the Kalman filter is described with Expressions (18) and (19), which depend on the following elements: $x_{k}$ is the state vector, ${\hat{x}}_{k}^{-}$ and ${\hat{x}}_{k}$ are the prior and posterior estimations of the state vector, $P_{k}^{-}$ and $P_{k}$ are the prior and posterior estimations of the state covariance matrix, F and H are the transition and measurement matrices, Q and R are the process and measurement noises covariance matrices, $K_{k}$ is the Kalman gain which corrects the vector prior estimation with the process innovation vector $y_{k}=z_{k}-H{\hat{x}}_{k}^{-}$, where $z_{k}$ is the noisy measurement. Expression (18) corresponds to the prediction phase, where an estimate of the current state variables is obtained:

(18) $$\begin{array}{cc} {\hat{x}}_{k}^{-} & =F{\hat{x}}_{k-1}\\ P_{k}^{-} & ={\mathrm{FP}}_{k-1}F^{T}+Q \end{array}$$

These estimates are updated using a weighted average of the next measurements, once observed.

(19) $$\begin{array}{cc} K_{k} & =P_{k}^{-}H^{T}{\left({\mathrm{HP}}_{k}^{-}H^{T}+R\right)}^{-1}\\ {\hat{x}}_{k} & ={\hat{x}}_{k}^{-}+K_{k}y_{k}\\ P_{k} & =\left(1-K_{k}H\right)P_{k}^{-} \end{array}$$

In order to use a Kalman filter for tracking moving targets, a dynamic model of the target motion must be designed. Since the intended targets are ground vehicles, motion in a plane with X and Y coordinates is considered, and a state vector is defined whose elements are the position, velocity, and acceleration of the target in both dimensions ($x^{T}=\left[x\;v_{x}\;a_{x}\;y\;v_{y}\;a_{y}\right]$, where *T* is the transpose operation). A quasi-constant acceleration model in both x and y dimensions is selected to describe the expected dynamics of the targets, the acceleration derivative being modelled as a white noise process with power spectral density *q* [26]. Therefore, the state function and state noise covariance matrices can be defined as $F=diag(F_{1})$ and $Q=diag(Q_{1})$, where $F_{1}$ and Q1 are the matrixes presented in (20).

(20) $$F_{1}=\left(\begin{array}{ccc} 1 & T & T^{2}\\ 0 & 1 & T\\ 0 & 0 & 1 \end{array}\right);\;\;Q_{1}=q\left(\begin{array}{ccc} T^{5}/20 & T^{4}/8 & T^{3}/6\\ T^{4}/8 & T^{3}/3 & T^{2}/2\\ T^{3}/6 & T^{2}/2 & T \end{array}\right)$$

Due to the linearity of the selected state function, the filter estimation stage is implemented with the standard Kalman filter equations (18). On the contrary, the geometrical considerations of the GPS-based PR presented in Section 2.1 give rise to non-linear relationships between the measurement vector $z_{k}={\left[m,p,\phi \right]}^{T}$ and the local Cartesian plane where the state vector $x_{k}$ is defined. For this reason, the suboptimal extended Kalman filter approach is selected to produce the filter correction stage expressed in (21), tackling the non-linearities of the measurement function *h* by a local linearisation [27].

(21) $$\begin{array}{cc} K_{k} & =P_{k}^{-}H^{T}{\left({\mathrm{HP}}_{k}^{-}H^{T}+V_{k}{\mathrm{RV}}_{k}^{T}\right)}^{-1}\\ x_{k} & =x_{k}^{-}+K_{k}(z_{k}-h\left({\hat{x}}_{k}^{-},0\right)\\ P_{k} & =\left(1-K_{k}H\right)P_{k}^{-} \end{array}$$

where, $K_{k}$ is the Kalman gain, R is the covariance matrix of the measurement error, and H and V are the Jacobian matrixes of the measurement function *h* with respect to the state vector and the measurement noise, respectively.

For tracker initialisation, target’s location and velocity are calculated following the procedure described in Section 3.4 and Section 3.5.

## 5. Simulation Results

**Figure 7:**
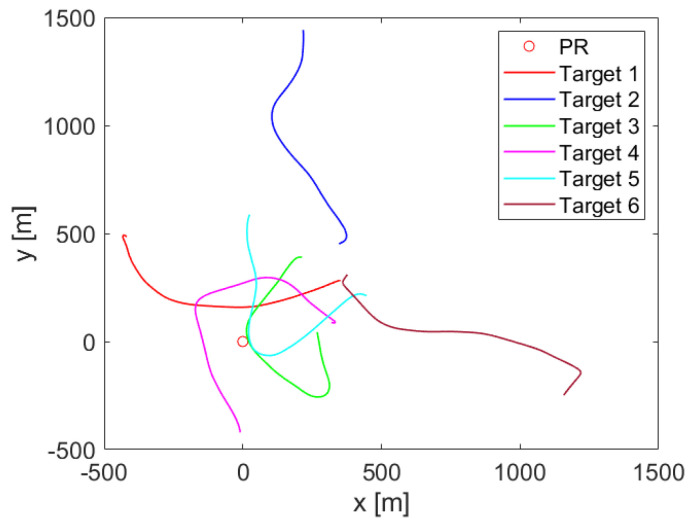
PR receiver location and targets’ trajectories in the simulated scenario.

The localization and tracking scheme was first validated with synthetic data. A simulated radar scenario was generated considering the variant geometry and signal characteristics of the GPS constellation. The PR receiver is considered at the center of the local Cartesian space where the simulation is performed. The PR receiver antennas were considered at 2 m from the ground level where the movement of the targets was performed following the trajectories represented in Figure 7. A GPS satellite was selected as the illumination source. Real satellite ephemerides were used to include the motion of the space platform in the simulated geometry. The initial location of the satellite was placed at an elevation of $35.54^{\circ }$ and an azimuth of $237.3^{\circ }$ from true North.

Six different targets with random initial positions around the PR location were simulated. A model with a quasi-constant modulus acceleration vector with independent components in X and Y coordinates was selected as the dynamic model of the targets. Initial velocities and accelerations were randomly selected from typical ground vehicle values.

The position and dynamics of the IoO and targets were recalculated every 250 ms, simulating the PR refresh rate. Bistatic range, Doppler shift, and azimuth measurements were calculated from each instantaneous radar geometry. Additive white Gaussian noise with standard deviations of $\sigma_{m}$ = 7.79 m, $\sigma_{p}$ = 1.39 Hz and $\sigma_{\phi }$ = 1.87${}^{\circ }$ for each respective measurement was selected to simulate the expected performance of GPS-based PR in real operation. A total of 160 time steps were simulated, resulting in a 40 s simulation. The simulated radar scenario geometry and target trajectories are depicted in Figure 7.

**Figure 8:**
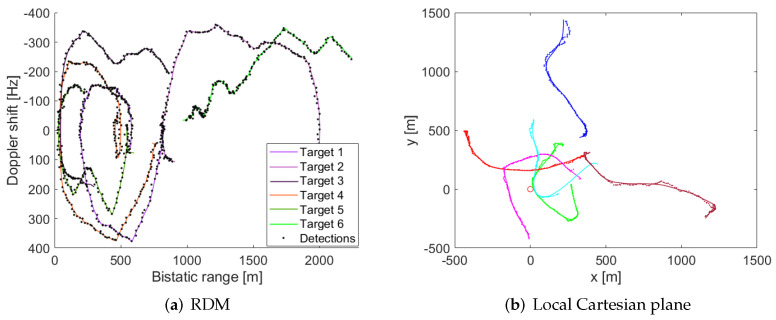
Results for the simulated radar scenario: (**a**) confirmed detections and associated tracks in the measurement space (range-Doppler); (**b**) Cartesian space tracking results (+ marker) and reference targets’ path presented in Figure 7 (solid line).

The localization and tracking results in the measurement and the tracking spaces for the simulated scenario are depicted in Figure 8. According to the RDM tracking results, a good trajectory estimation is obtained with reduced measurement errors. When the results are translated to the Cartesian plane, correct system performance is shown for most of the simulated trajectories, with higher localization errors after tight turns. However, the inclusion of new detections results in the convergence of the trajectory to the actual target location. Moreover, the longer distance to the PR location results in higher errors, being more relevant in the final part of the trajectory of the second target. Finally, a loss of tracking occurs at the beginning of the trajectory of target 5 due to the mixture of detections of different targets at nearby locations resulting in an unexpected track movement and thus the way out of the target from the detection gate. However, a new track is automatically generated and the remaining trajectory is correctly estimated.

**Figure 9:**
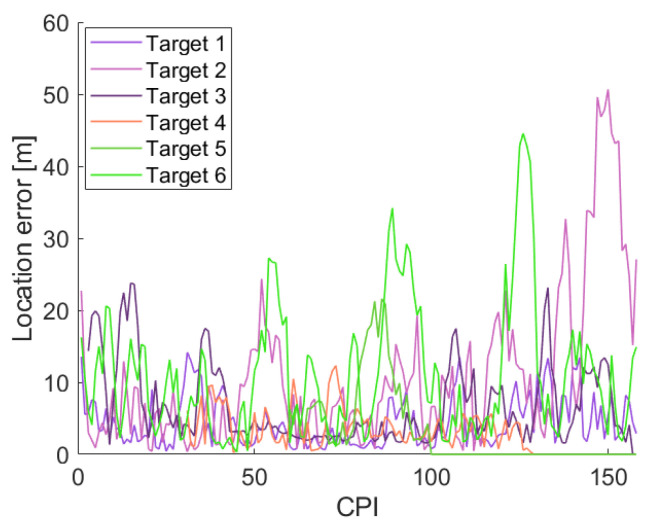
Localization error in the simulated scenario.

The localization error was also calculated as the Euclidean distance between the estimated track and the actual target location. Figure 9 shows the estimated localization error over the simulation time for the six targets. As expected from Figure 8b, the localization error remains low along most of the tracked trajectories, with larger localization errors appearing at tight turns of the targets and when the distance between the target and the PR increases. However, despite the localization error can be as higher as 50 m at target 2, the average localization error of each simulated target remains low: 4.44 m, 10.76 m, 6.65 m, 2.96 m, 3.09 m, and 11.19 m, respectively.

## 6. Results with Real Data

### 6.1. Radar Scenario

**Figure 10:**
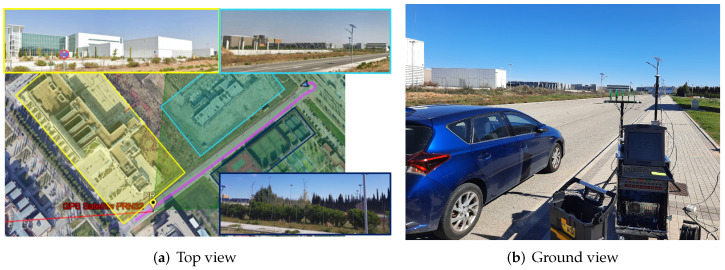
Trials radar scenario: (**a**) top view with PR location, satellite direction (PRN32), area of interest (green area), cooperative target trajectory from GPS data (purple line), buildings and sport facilities details are shown in pictures with their associated area; (**b**) ground view of the AoI, cooperative vehicle and PR receiver deployment.

A real radar data acquisition campaign was carried out to evaluate the ground target detection and localization performance of the proposed GPS-based PR. An area of the external campus of the University of Alcalá, near the Faculty of Physiotherapy and Nursing, was selected as the radar scenario. Figure 10a shows a Google Earth image of the Area of Interest (AoI). The radar scenario is composed of a 390-m straight road with several buildings and sports facilities at both sides. The biggest building is mainly made of metal (shown in Figure 10 in the yellow area), and the numerous metal fences and high lampposts distributed throughout the sports facilities. During the experiments, the PR receiver was placed on the pavement near the road and a cooperative vehicle was driven along the road starting its movement from the back of the PR location. The PR receiver deployment and the cooperative target are depicted in Figure 10b.

An updated version of the IDEPAR technological demonstrator, developed at the University of Alcalá [17] was employed to acquire the real radar data. The PR demonstrator was composed of a commercial active GPS antenna, right hand circularly polarised with 3 + 30 dB gain and hemispherical pattern, for the reference channel acquisition. The surveillance channel was composed of a uniform five-element linear array whose individual elements were linearly polarised antennas with an azimuthal beamwidth of 80 degrees and a gain of 6.8 dB.

For the tests, the GPS satellite constellation was selected as IoOs, and the civil GPS signal in the L1 band was selected as the illumination signal. The PR demonstrator was configured to acquire 40 s of continuous signal at GPS L1 band (1575.42 MHz). The illumination signal was oversampled up to an acquisition rate of 25 MHz to increase the bistatic range accuracy and to allow ground target localization at low ranges. The acquired data were divided into 160 non-overlapping Coherent Processing Intervals (CPI), each of 250 ms duration, that were processed following the scheme described in Section 2 and Section 3. For the first stage of the spatial filtering, 3 different orthogonal beams were generated (−25${}^{\circ }$, 0${}^{\circ }$, 25${}^{\circ }$), the set of simultaneous beams was complemented with two additional beams at the first intersections of the orthogonal ones (−15${}^{\circ }$, 15${}^{\circ }$).

A search of available GPS satellites was performed, identifying seven satellites, with PRNs 2, 6, 12, 25, 29, 31, and 32. The GPS signals associated with these PRN codes were reconstructed and considered in the adaptive filtering process to reduce their interference contributions in the RDMs. The satellite transmitting PRN 32 was selected as IoO due to its higher power at ground level.

Some tests have been performed on the radar scenario with moving cooperative targets to evaluate the performance of the system. The cooperative targets started from the rear of the passive radar. A straight road trajectory of 390 m was defined, with the targets moving at a nearly constant speed of 30 km/h along the trajectory on the AoI. Nevertheless, the variation of the bistatic geometry produced an accelerated motion in the bistatic range-Doppler domain.

### 6.2. Results

In this subsection, some results of ground target detection with the PR using GPS satellites as IoO are presented to demonstrate the actual system performance in the radar scenario.

**Figure 11:**
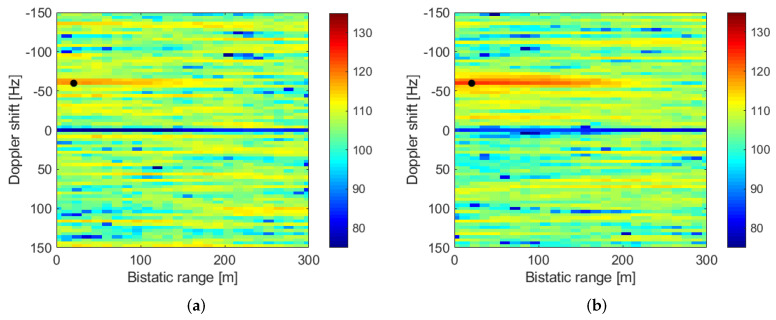
Real radar data results: first stage spatial filtering. (**a**) RDM from single array element. (**b**) RDM after beamforming (12.5${}^{\circ }$ beam).

The first result to be described correspond to the detection of a cooperative target in the radar scenario. Figure 11a plots the RDM for the first single element of the surveillance array at CPI = 9. At this instant, the target echo was at 24 m bistatic range and the Doppler shift was −60 Hz, with a CAF level of 118 dB. The target echo shows a range extension with a starting point outside the CAF range limits and an end point at approximately 100 m. This echo extension is explained by the difference between the GPS signal bandwidth (2 MHz) and sampling frequency (25 MHz), and the size of the range bins (12 m).

Figure 11b shows the RDM when the first step spatial filtering is applied, for the beam in which the higher target return is obtained (beam pointing to 12.5${}^{\circ }$, because the target was expected at 10${}^{\circ }$). The target’s echo level increases by approximately 6.5 dB, up to 125.64 dB. To obtain a more accurate estimation of the target range, the ambiguity function was approximated to a parabola, using 5 level points of the RDM around the echo’s maximum. The range at which the maximum of the interpolating function is obtained, is the estimated target location, equal to 18.48 m for this time instant, whereas the target’s range obtained from its GPS data was 20.60 m.

**Figure 12:**
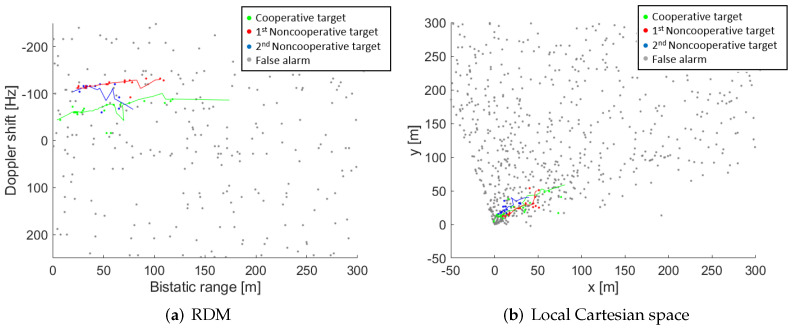
Real radar data results: Cumulative detection output in measurement and tracking domain. (**a**) RDM. (**b**) Local Cartesian space.

Figure 12 plots the complete detection results, in both the measurement and the tracking space, with one cooperative target, and two non-cooperative targets in the AoI (the results corresponding to the cooperative target are highlighted in green colour). Because the selected road of interest belongs to the university campus and its traffic density is low, only 2 non-cooperative targets performed movements during the acquisition time. The results of detection and tracking of the non-cooperative targets are depicted in Figure 12 with blue and red colours. The PR configuration allows detection of all targets up to a maximum bi-static distance of 118 m (Figure 12a). However, the presence of the non-cooperative targets only in the last part of the acquisition slightly reduces the number of detection points. The cooperative target was detected at 24 CPIs at the beginning of the acquisition, while the non-cooperative targets were detected at 11 and 19 CPIs, and were no longer detected at the end of the acquisition.

Figure 12b shows the targets’ location estimation on the local Cartesian space centred in the PR receiver and with the North direction following the *y*-axis. As it was expected from targets’ detections in the RDMs, the cooperative target reaches the higher detection distances from PR location (98 m) due to its movement was performed during the beginning of the acquisition and overpassed the detection coverage. The detection range for non-cooperative targets remains lower (68 m and 55 m) due to their movements along the AoI were carried out at the end of the measurement, and could not overpass the detection coverage before the end of the acquisition.

**Figure 13:**
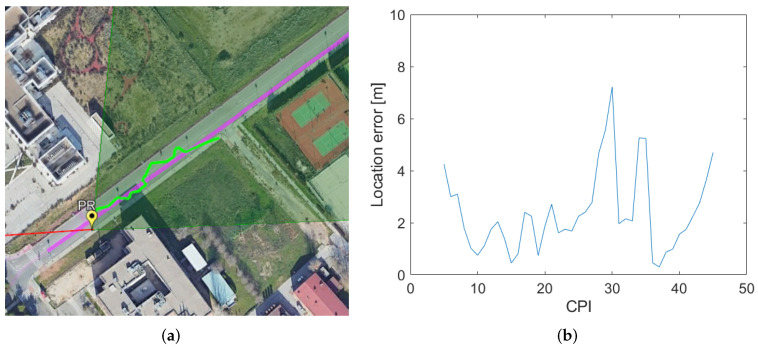
Localization results for a cooperative target: (**a**) Google Earth view of the cooperative target’s estimated position (green) versus target GPS data (purple); (**b**) Estimated localization error for the cooperative target.

The targets’ location estimations in the local Cartesian spaces were transformed to the geodetic coordinates for easy integration into the Google Earth application. Figure 13a depicted the Google Earth view of the localization results obtained for the cooperative target in comparison with the target’s trajectory from GPS data in the radar scenario. The estimated trajectory is mostly on the road, close to the actual location provided by the GPS data of the cooperative target. The higher estimation deviations are presented in the second part of the trajectory where the target’s SNR decreases and detection failures appear. Target detection and localization start when the target trajectory exceeds the PR position and continues for more than 10 s, reaching a maximum detection distance of 98 m from the PR location.

System localization capability has also been analysed, taking into account the Euclidean distance between the estimated and the actual location provided by the GPS data of the target. Figure 13b shows the estimated localization error for the cooperative target as a function of the CPIs at which the target estimation was available. The localization error remains below 8 m along the entire trajectory, with the highest deviations in the second part of the trajectory where the target detection probability reduces. The average localization error is 2.38 m. Therefore, the combination of GPS-based PR target detection from a single satellite transmitter and the application of beamforming techniques for localization in a local Cartesian space results in high quality localization performance even though the bistatic resolution associated with the selected GPS signal (2 MHz bandwidth) is 150 m.

**Figure 14:**
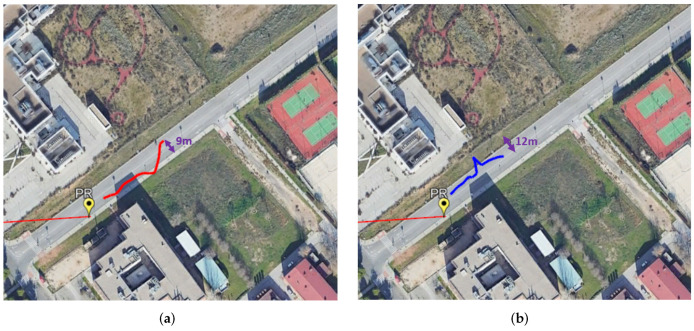
Localization results for non-cooperative targets moving away from PR location (blue and red). (**a**) First non-cooperative target. (**b**) Second non-cooperative target.

Results obtained for both non-cooperative targets are shown in Figure 14. As in the case of the cooperative target, their estimated trajectories mostly fit the road line for their direction of motion (right side of the road away from the PR location). The highest deviations are obtained for the second non-cooperative target, with a maximum error of 12 m from its road line whereas the first non-cooperative target’s maximum deviation from its road line is approximately 9m. Targets detection starts once their movement reaches the coverage of the surveillance antennas. In this case, the maximum detected distances are approximately 68 and 55 m from the PR location for the first and second non-cooperative targets, respectively. Because both non-cooperative targets appear at the final part of the acquisition, their associated trajectories are shorter than the cooperative one. The lack of GPS data from these targets does not allow the real estimation of the localization error along their trajectory.

A summary of the location statistics (mean μ and standard deviation σ) obtained for the estimations in both synthetic and real data are presented in Table 1. The better location results are obtained for the cooperative target on the real radar scenario due to its movement close to the PR location. The location error is highly dependent on PR to target distance giving rise to worse location performances for simulated targets far from PR location. Nevertheless, system performances in overall studied cases confirm the applicability of the proposed processing scheme.

The assumption of a known target altitude limits the use of the proposed processing scheme to maritime and terrestrial targets. The reflectivity of maritime targets is usually higher, which allows the detection at higher distances. Since the localization error depends directly on the target’s range when only the DoA information is exploited, the absolute error in target localization is expected to be larger. To address both the increase of localization errors with target distance and the position of airborne targets, future works will study solutions based on multi-static configurations exploiting several line-of-sight GPS transmitters.

## 7. Conclusions

This paper presents a GPS-based PR exploiting digital array processing techniques for ground target detection and localization. To cope with problems related to the temporal variation and low power of the received signal at ground level, a processing scheme combining reference signal reconstruction, adaptive filtering techniques, and a two-stage spatial filtering algorithm is implemented. Conventional beamforming techniques are used to increase the echo level of the target prior to the detection stage. A high-resolution DoA estimation technique is applied to estimate the target azimuth and allow the target to be located on the ground, first in local Cartesian space and then in geodetic coordinates.

Real radar data were recorded using an updated version of IDEPAR demonstrator at the external campus of the University of Alcalá, in order to analyse the system detection and localization performance. During the measurement campaign, a cooperative vehicle was used for validation purposes and two additional non-cooperative targets were available in the AoI. The use of a linear array in the surveillance channel increased the target’s echo level improving the system detection capabilities and the maximum detection range. The target localization capability was validated along the trajectory of the cooperative targets with an average estimation error of 2.38 m. Non-cooperative targets were also correctly located along their trajectories. The results confirm the possibility of detecting ground targets with GPS-based PR, but at low ranges currently. Further research is necessary to increase the detection range, maintaining the positive characteristics of these radars. 

## Figures and Tables

**Table 1:** Location error statistics for simulated and real GPS based PR data.

	Simulated Data							Real Data
	Targ 1	Targ 2	Targ 3	Targ 4	Targ 5	Targ 6	Total	Coop. Targ
μ [m]	4.44	10.76	6.65	2.96	3.09	11.19	7.46	2.38
σ [m]	3.5	10.76	5.57	7.86	5.38	9.31	7.77	1.58
